# Inulae Flos and Its Compounds Inhibit TNF-**α**- and IFN-**γ**-Induced Chemokine Production in HaCaT Human Keratinocytes

**DOI:** 10.1155/2012/280351

**Published:** 2012-08-07

**Authors:** Jung-Hoon Kim, Hye-Sun Lim, Hyekyung Ha, Chang-Seob Seo, Hyeun-Kyoo Shin

**Affiliations:** Basic Herbal Medicine Research Group, Korea Institute of Oriental Medicine, Daejeon 305-811, Republic of Korea

## Abstract

The present study is to investigate which kinds of solvent extracts of Inulae Flos inhibit the chemokine productions in HaCaT cell and whether the inhibitory capacity of Inulae Flos is related with constitutional compounds. The 70% methanol extract showed comparatively higher inhibition of thymus and activation-regulated chemokine (TARC/CCL17) in HaCaT cells, therefore this extract was further partitioned with n-hexane, chloroform, ethyl acetate, butanol, and water. The ethyl acetate fraction inhibited TARC, macrophage-derived chemokine (MDC/CCL22), and regulated on activation of normal T-cell-expressed and -secreted (RANTES/CCL5) production in HaCaT cells better than the other fractions. The compounds of Inulae Flos, such as 1,5-dicaffeoylquinic acid and luteolin, inhibited TARC, MDC, and RANTES production in HaCaT cells. 1,5-Dicaffeoylquinic acid was contained at the highest concentrations both in the 70% methanol extract and ethyl acetate fraction and inhibited the secretion of chemokines dose-dependently more than the other compounds. Luteolin also represented dose-dependent inhibition on chemokine productions although it was contained at lower levels in 70% methanol extract and solvent fractions. These results suggest that the inhibitory effects of Inulae Flos on chemokine production in HaCaT cell could be related with constituent compounds contained, especially 1,5-dicaffeoylquinic acid and luteolin.

## 1. Introduction

Inulae Flos, the inflorescence of *Inula japonica* or *I. britannica* (Asteraceae), has demonstrated therapeutic efficacy by reducing phlegm, promoting the dissipation of pathological water, redirecting the qi downward, and stopping vomiting. The therapeutic efficacy of Inulae Flos has prompted its use in the treatment of symptoms such as the accumulation of phlegm and fluids clogging up the lungs, vomiting, hiccough, belching, and cough with excessive expectoration of phlegm [1].

Recent pharmacological studies of Inulae Flos have shown hepatoprotective [2], immunoregulatory [3], antidiabetic [4], hypolipidemic [4], anticancer [5], antiinflammatory [6], antioxidant, and neuroprotective properties [7] when it was evaluated as water or organic solvent extracts of the whole herbal medicine. Its pharmacological activity has been associated not only with the whole herbal medicinal extract but also with compounds extracted from the herbal medicine. Although crude extracts of a single herbal medicine or herbal formula can exhibit striking biological effects, their mechanisms cannot be fully established because innumerable compounds are contained in even a single herbal medicine.

Most studies of the biological effects or mechanisms of herbal medicines concentrate on the main compound of the herbal medicine. Compounds isolated from Inulae Flos have shown pharmacological activities, such as iNOS inhibition by 1-O-acetyl-4R,6S-britannilactone [8], the antitumour effects of sesquiterpenelactones [9], the antidiabetic effects of polysaccharides [10], the antioxidative effects of flavonoids [11], and the inhibition of NO production by sesquiterpenes [12].

The compounds contained in herbal medicine can be identified with analytical techniques, and the predominant compound is often thought to be strongly associated with the biological effect. The chemical compounds in Inulae Flos were analysed with high-performance liquid chromatography-ultraviolet detection (HPLC-UV), as reported in previous papers, and the structures of the flavonoids and sesquiterpenes were determined [13, 14].

In the present study, we extracted Inulae Flos with different solvent compositions then further partitioned the extracts to determine the constituent having the predominant biological effect. The concentrations of eight compounds of Inulae Flos were quantified in extract by different solvent compositions and solvent fractions to determine the relationships between the inhibitory effect of Inulae Flos and its constituent compounds on chemokine productions in HaCaT cell.

## 2. Materials and Method

### 2.1. Reagents and Plant Materials

**Figure 1:**
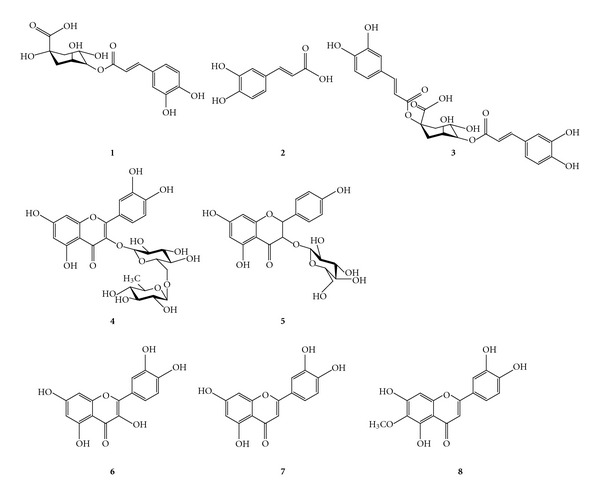
Chemical structures of the components of Inulae Flos. Chlorogenic acid (**1**), caffeic acid (**2**), 1,5-dicaffeoylquinic acid (**3**), rutin (**4**), kaempferol-3-O-glucoside (**5**), quercetin (**6**), luteolin (**7**), and 6-methoxy-luteolin (**8**).

HPLC-grade methanol, ethanol, acetonitrile, and water were purchased from J. T. Baker Inc. (Phillipsburg, NJ, USA). Caffeic acid (99%) and chlorogenic acid (99%) were purchased from Acros Organics (NJ, USA). Rutin (95%), quercetin (98%), and luteolin (99%) were obtained from Sigma-Aldrich (St Louis, MO, USA). 6-Methoxy-luteolin and kaempferol-3-O-glucoside were purchased from ChromaDex (Irvine, CA, USA) and 1,5-dicaffeoylquinic acid (99.2%) from Chengdu Biopurify Phytochemicals (Chengdu, China). The chemical structures of the standard compounds were classified as phenylpropanoids and flavonoids, as shown in Figure 1. Inulae Flos was obtained from local market of herbal medicine (Kwangmyungdang Medicinal Herbs, Ulsan, Republic of Korea). A voucher specimen (ST2011-13) was deposited in the Basic Herbal Medicine Research Group of the Korea Institute of Oriental Medicine.

### 2.2. Extraction of the Herbal Medicine

The dried aerial part of Inulae Flos (1.0 g) was pulverized through a 60 mesh sieve and extracted with 100 mL of 70% (v/v) methanol, 70% (v/v) ethanol, 100% methanol, 100% ethanol, and deionized water for 60 min with sonication, respectively. Each extract was filtered through a SmartPor GHP syringe filter (Woongi Science, Seoul, Korea) before it was injected into the HPLC apparatus. The remaining extracts were filtered through a paper filter (Advantec, Japan) and concentrated with a rotary evaporator under vacuum for biological testing. The yields of the extracts were 10.05% in 70% methanol, 11.93% in 70% ethanol, 7.29% in 100% methanol, 4.04% in 100% ethanol, and 12.51% in deionized water.

### 2.3. Partitioning of the Solvent Extracts

The 70% MeOH extract of powdered Inulae Flos (145 g) was suspended in water and further partitioned successively with n-hexane, chloroform, ethyl acetate, and butanol. Each solvent fraction was filtered through a paper filter and concentrated with a rotary evaporator under vacuum for biological testing. The dried extracts were dissolved in methanol to a concentration of 1000 ppm and filtered through a syringe filter for HPLC analysis.

### 2.4. Preparation of Standard Solutions

Accurately weighed standard compounds were dissolved in methanol to produce stock solutions at concentrations of 1 mg/mL. The stock solution containing a standard compound was diluted to make working solutions, which were used to construct a calibration curve.

### 2.5. Chromatographic Instrumentation and Conditions

The HPLC system used was a Shimadzu LC-20A (Kyoto, Japan) equipped with a solvent delivery unit (LC-20AT), an autosampler (SIL-20AC), column oven (CTO-20A), degasser (DGU-20A_3_), and photodiode array detector (SPD-M20A). Separation was performed on a Gemini C_18_ column (4.6 × 250 mm, 5 *μ*m; Phenomenex, Torrance, CA, USA). The mobile phase consisted of water containing 1% acetic acid (A) and acetonitrile (B). The composition of the mobile phase was 20%–40% (B) in 0–15 min, held for 35 min, and 40%–100% (B) in 50–55 min, held for 5 min. The column temperature was maintained at 40°C. The flow rate was 1.0 mL/min, and the injection volume was 10 *μ*L. All standards and samples were detected at wavelengths of 255, 325, and 340 nm.

### 2.6. Precision and Recovery

The intra- and interday precision was calculated by analysing a sample extracts spiked with three different concentrations levels of reference compounds (low, medium, and high). The relative standard deviation (RSD) was measured in three replicates of the spiked samples to assess the intra-day precision and in three days to assess the interday precision. Recovery was tested by adding three different concentrations levels of reference compounds (low, medium, and high) to the samples before extraction. The methods described above were used to extract and analyse the compounds. The recovery was calculated as follows: Recovery (%) = ((detected concentration − original concentration)/spiked concentration) × 100.

### 2.7. Cell Culture

Human keratinocyte cell line HaCaT was kindly provided from Dr. Na Gyong Lee (Sejong University, Seoul, Republic of Korea). HaCaT cells were cultured in Dulbecco's modified Eagle's medium (Gibco Inc., NY, USA) supplemented with 10% heat-inactivated foetal bovine serum (Gibco Inc.), penicillin (100 U/mL), and streptomycin (100 *μ*g/mL) in a 5% CO_2_ incubator at 37°C.

### 2.8. Cytotoxicity Assay

Cell viability was assessed with the CCK-8 assay (Cell Counting Kit-8 from Dojindo, Kumamoto, Japan) according to the manufacturer's instructions. HaCaT cells (1 × 10^3^ cell/well) were incubated in 96-well plates with various concentrations of the test materials for 24 h. CCK-8 reagent was added to each well and incubated for 4 h. The absorbance was measured at 450 nm with a Benchmarkplus microplate reader (Bio-Rad Laboratories, Hercules, CA, USA). The percentage of cell viability was calculated with the following formula: cell viability (%) = (mean absorbance in test wells/mean absorbance in control wells) × 100.

### 2.9. Measurement of Chemokine Production

HaCaT cells (1 × 10^6^ cell/well) were cultured in six-well plates in medium containing 10% foetal bovine serum. After having reached confluence, the cells were washed and incubated with 1 mL of serum-free medium containing tumour necrosis factor-*α* (TNF-*α*) and interferon-*γ* (IFN-*γ*; each 10 ng/mL; R&D Systems Inc., Minneapolis, MN, USA) for 24 h to stimulate the cells. The supernatants of the cells were harvested, and the productions of TARC, MDC, and RANTES were quantified using an enzyme-linked immunosorbent assay (ELISA), performed according to the protocol provided by R&D Systems.

### 2.10. Statistical Analysis

All experiments were performed at least three times. One-way analysis of variance was used to identify significant differences between the treatment groups. Dunnett's test was used for multigroup comparisons. Differences were considered significant at *P* < 0.05 or *P* < 0.01.

## 3. Results

### 3.1. Linear Regression, Limit of Detection (LOD), and Limit of Quantification (LOQ)

**Figure 2:**
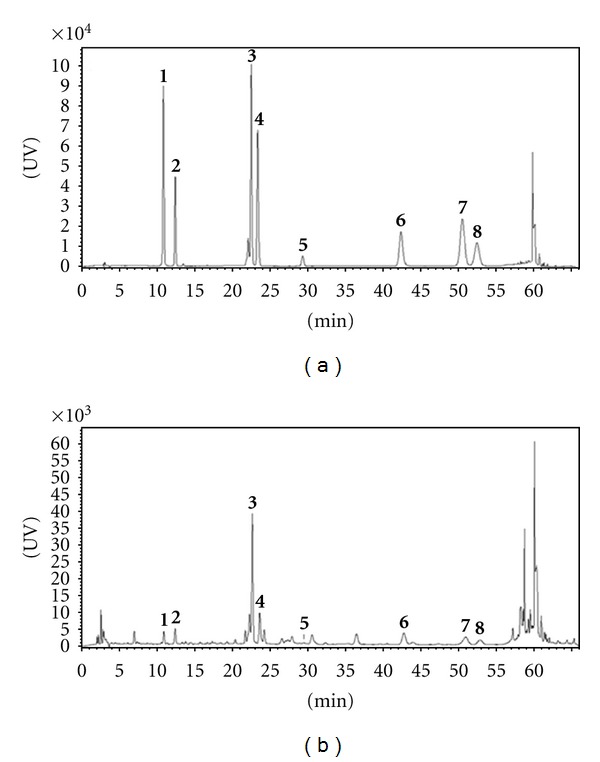
HPLC chromatograms of a standard mixture (a) and a 70% methanol extract of Inulae Flos (b). Chlorogenic acid **(1)**, caffeic acid **(2)**, 1,5-dicaffeoylquinic acid **(3)**, rutin **(4)**, kaempferol-3-O-glucoside **(5)**, quercetin **(6)**, luteolin **(7)**, and 6-methoxy-luteolin **(8)**.

Accurately weighed standard compounds were dissolved in methanol and diluted to six levels of concentrations to construct calibration curves. The correlation coefficient (*r* ^2^) for each compound ranged from 0.9995 to 0.9999 which showed good linearity. The LODs and LOQs were calculated at the concentrations of each compound that produced signal-to-noise ratios of 3 and 10; their values were LOD = 0.02–0.13 *μ*g/mL and LOQ = 0.06–0.43 *μ*g/mL (Table 1). All compounds were detected in the sample extracts and were well separated on chromatograms with the methods described above (Figure 2).

### 3.2. Precision and Recovery

The precision of each standard compound was evaluated as relative standard deviation (RSD), calculated as the percentage of standard deviation divided by the mean value. The RSD values for the intra-day and interday precision were 0.23%–3.24% and 0.04%–2.60%, respectively, (Table 2). Recovery was used to test the accuracy of the experimental method. The recovery of each standard compound was in the range of 93.09%–111.13%, with an RSD of less than 3.0% (Table 3).

### 3.3. Effects of the Test Materials on Cell Viability

To determine the cytotoxicity of the test materials on HaCaT keratinocytes, the cells were exposed to various concentrations of the extracts and single compounds for 24 h. Cell viability was then measured using the CCK-8 assay. The nontoxic concentrations of the test materials were used for the subsequent experiments (data not shown).

### 3.4. Constituent Reference Compounds in Inulae Flos Extract Determined in Different Solvent Compositions and Their Effects on TARC Expression in Cells Treated with TNF-*α* and IFN-*γ*

To determine and select the extract showing the optimum solvent composition, a quantitative analysis was performed with extract by different solvents. The extracts produced with aqueous alcohol (70% methanol or 70% ethanol), absolute alcohol (100% methanol or 100% ethanol), or water contained different proportions of the reference compounds. Higher levels of phenylpropanoid-structured compounds, including chlorogenic acid and 1,5-dicaffeoylquinic acid, were found in the aqueous alcohol extracts than in the other solvent extracts, except for caffeic acid, of which the content was higher in the water extract. The contents of the flavonoid-structured compounds, including rutin, kaempferol-3-O-glucoside, quercetin, luteolin, and 6-methoxy-luteolin, were the highest in the alcohol extracts, except for rutin contained the highest level in the ethanol extract. The water extract showed a markedly higher content of caffeic acid than any other solvent extract, and slightly more 1,5-dicaffeoylquinic acid was found in the water extract than in the ethanol extracts (Table 4). The predominant compound on the HPLC chromatograms, 1,5-dicaffeoylquinic acid, was higher content than other compounds in all the solvent compositions of which the content in 70% methanol extract was most abundant.

**Figure 3:**
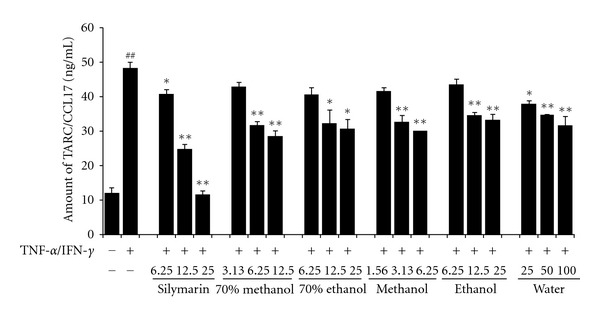
Effects of Inulae Flos extract on TARC/CCL17 production in HaCaT cells. Cells were treated with various Inulae Flos extracts (70% methanol, 6.25–25 *μ*g/mL; 70% ethanol, 6.25–25 *μ*g/mL; 100% methanol, 3.13–12.5 *μ*g/mL; 100% ethanol, 1.56–6.25 *μ*g/mL; water, 25–100 *μ*g/mL) and then costimulated with TNF-*α* and IFN-*γ* (each 10 ng/mL) for 24 h. As the positive control, cells were treated with silymarin (6.25–25 *μ*g/mL). The levels of TARC released into the culture medium were assessed using a commercially available ELISA kit. Each bar represents the mean of three independent experiments. ^##^ *P* < 0.01 versus vehicle-treated control group; *P < 0.05 and **P < 0.01 versus TNF-*α*/IFN-*γ*-treated cells.

The effect of the solvent composition was determined by comparing the inhibitory effects of each extract according to its solvent compositions. As shown in Figure 3, HaCaT cells treated with TNF-*α*/IFN-*γ* (TI) expressed significantly higher TARC level (48.2 ± 1.80 ng/mL, *P* < 0.01) than the controls (12.1 ± 1.40 ng/mL). In contrast, the silymarin-treated groups showed significant reductions in TARC level (40.7 ± 1.35 ng/mL in 6.25 *μ*g/mL; 24.7 ± 1.44 ng/mL in 12.5 *μ*g/mL; 11.7 ± 0.99 ng/mL in 25 *μ*g/mL) compared with the TI-treated cells. The 70% methanol extract (31.66 ± 1.17 ng/mL in 12.5 *μ*g/mL; 28.60 ± 1.60 ng/mL in 25 *μ*g/mL) significantly reduced the level of TARC in a dose-dependent manner compared with the level in the TI-treated cells although the other extracts of Inulae Flos, including the 70% ethanol, 100% methanol, 100% ethanol, and water extracts also showed reductions in TARC compared with that in the TI-treated cells.

Based on the rate of inhibition of TARC release and the half maximal inhibitory concentration (IC_50_) of each extract, the 70% methanol extract (IC_50_ = 16.1 *μ*g/mL) was selected as the test extract for subsequent experiments because of more inhibitory capacity than the other solvents, and its effects on the release of chemokines, including TARC, RANTES, and MDC, from HaCaT cells were investigated.

### 3.5. Content of the Reference Compounds and the effects of 70% Methanol Fractions on the TNF-*α*- and IFN-*γ*-Induced Chemokine Release from HaCaT Cells

Five fractions of the 70% methanol extract were obtained and analysed quantitatively to investigate the contents of the compounds in each solvent fraction and to determine whether different contents of the compounds affected the biological effect. The ethyl acetate fraction showed the highest contents of caffeic acid, 1,5-dicaffeoylquinic acid, rutin, kaempferol-3-O-glucoside, quercetin, luteolin, and 6-methoxy-luteolin. Only chlorogenic acid was highest content in the butanol fraction. The n-hexane fraction contained few compounds other than low levels of rutin. Although some compounds were found in the chloroform and butanol fractions, their levels and diversity were lower than those in the ethyl acetate fraction. The water fraction contained only phenylpropanoid-structured compounds, including chlorogenic acid, caffeic acid, and 1,5-dicaffeoylquinic acid, but no flavonoid-structured compounds (Table 5). 

**Figure 4:**
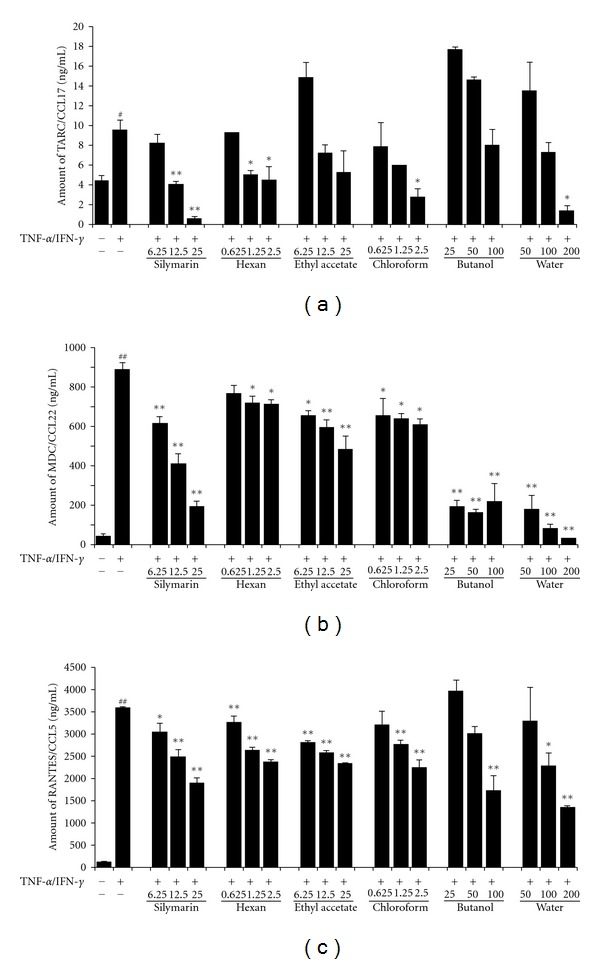
Effects of solvent fractions of the 70% methanol extract of Inulae Flos on chemokine production in HaCaT cells. Cells were treated with the different solvent fractions (hexane, 0.625–2.5 *μ*g/mL; ethyl acetate, 6.25–25 *μ*g/mL; chloroform, 0.625–2.5 *μ*g/mL; butyl alcohol, 25–100 *μ*g/mL; water, 50–200 *μ*g/mL) and then costimulated TNF-*α* and IFN-*γ* (each 10 ng/mL) for 24 h. As the positive control, cells were treated with silymarin (6.25–25 *μ*g/mL). The levels of TARC (a), MDC (b), and RANTES (c) released into the culture medium were assessed using commercially available ELISA kits. Each bar represents the mean of three independent experiments. ^##^ *P* < 0.01 versus vehicle control group; *P < 0.05 and **P < 0.01 versus TNF-*α*/IFN-*γ* treated cells.

The fractions of 70% methanol extract were tested to determine whether each fraction inhibited the productions of chemokines in HaCaT cells after the cells were treated with TNF-*α* and IFN-*γ*. TARC, MDC, and RANTES production in the TI-treated cells increased 2-, 20-, and 26-fold compared with that in the control cells, whereas the cells treated with most of fractions of 70% methanol extract reduced TARC, MDC, and RNATES levels compared with the TI-treated cells (Figure 4). As though the other fractions of 70% methanol extract, including the n-hexane, chloroform, butanol, and water fractions, also produced significant reductions in MDC and RANTES compared with that in the TI-treated cells, their inhibition rates were lower than that of the ethyl acetate fraction. As shown in Figures 4(b) and 4(c), the cells treated with the ethyl acetate fraction showed significantly reduced levels of MDC (657.37 ± 23.86 ng/mL in 6.25 *μ*g/mL; 593.81 ± 38.42 ng/mL in 12.5 *μ*g/mL; 483.02 ± 66.15 ng/mL in 25 *μ*g/mL) and RANTES (2820.00 ± 17.75 ng/mL in 6.25 *μ*g/mL; 2580.01 ± 38.64 ng/mL in 12.5 *μ*g/mL; 2343.45 ± 41.35 ng/mL in 25 *μ*g/mL) compared with the TI-treated cells, consistent with the results for silymarin.

### 3.6. Effects of Chemical Compounds on the Production of Chemokine Induced by TNF-*α* and IFN-*γ* in HaCaT Cells

**Figure 5:**
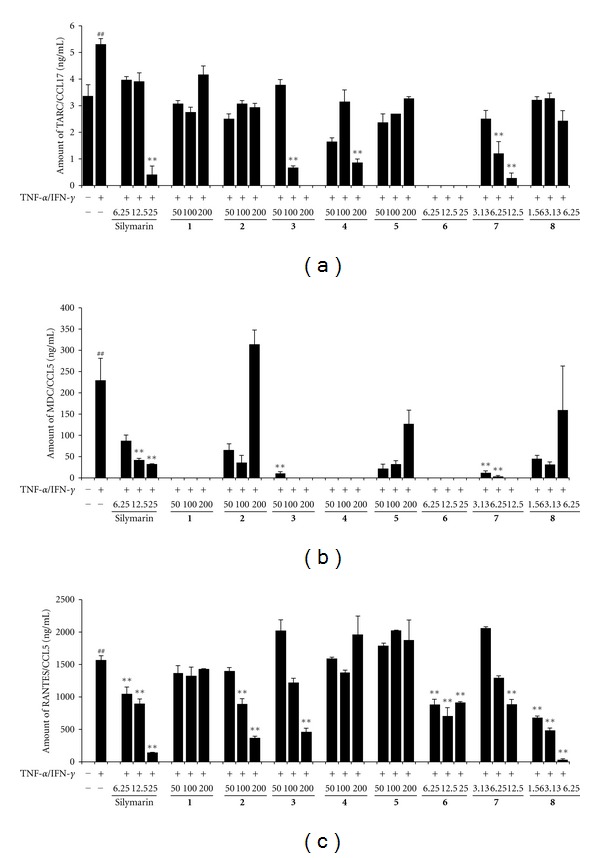
Effects of single compounds from Inulae Flos on chemokine production in HaCaT cells. Cells were treated with single compounds (**1**, 50–200 *μ*M chlorogenic acid; **2**, 50–200 *μ*M caffeic acid; **3**, 50–200 *μ*M 1,5-dicaffeoylquinic acid; **4**, 50–200 *μ*M rutin; **5**, 50–200 *μ*M kaempferol-3-O-glucoside; **6**, 6.25–25 *μ*M quercetin; **7**, 3.13–12.5 *μ*M luteolin; **8**, 1.56–6.25 *μ*M 6-methoxy-luteolin) and then costimulated with TNF-*α* and IFN-*γ* (each 10 ng/mL) for 24 h. As the positive control, cells were treated with silymarin (6.25–25 *μ*g/mL). The levels of TARC (a), MDC (b), and RANTES (c) released into the culture medium were assessed using commercially available ELISA kits. Each bar represents the mean of three independent experiments. ^##^ *P* < 0.01 versus vehicle-treated control group; *P < 0.05 and **P < 0.01 versus TNF-*α*/IFN-*γ*-treated cells.

When HaCaT cells were treated with TI for 24 h, TARC (5.31 ±0.21 ng/mL, *P* < 0.01) levels increased 1.6-fold compared with the vehicle-treated control group (3.38 ± 0.43 ng/mL). However, in 1,5-dicaffeoylquinic acid and luteolin-treated cell, TARC production was significantly inhibited in a dose-dependent manner (*P* < 0.01) (Figure 5(a)). MDC (229.57 ± 51.27 ng/mL) production increased compared with the vehicle-treated control group and its level was significantly reduced after 1,5-dicaffeoylquinic acid or luteolin treatment (*P* < 0.01) (Figure 5(b)). TI-treated cells showed significantly increased production of RANTES (1573 ± 68.99 ng/mL) relative to that in the control cells. These increases were inhibited dose-dependently by caffeic acid (893.47 ± 79.99 ng/mL in 100 *μ*g/mL; 374.35 ± 25.07 ng/mL in 200 *μ*g/mL, **2**), 1,5-dicaffeoylquinic acid (469.46 ± 53.44 ng/mL in 200 *μ*g/mL, **3**), luteolin (893.48 ± 71.99 ng/mL in 12.5 *μ*g/mL, **7**), and 6-methoxy luteolin (689.00 ± 20.13 ng/mL in 1.56 *μ*g/mL; 494.23 ± 28.68 ng/mL in 3.13 *μ*g/mL; 38.57 ± 15.92 ng/mL in 6.25 *μ*g/mL, **8**). However, other compounds examined did not significantly reduce the expression of RANTES in the TI-treated cells (Figure 5(c)).

## 4. Discussion

The HaCaT cell line is a human keratinocyte line that releases abnormal level of chemokines, including TARC, MDC, RANTES, vascular endothelial growth factor, and eotaxin when stimulated with TNF-*α* and IFN-*γ*. When released from keratinocytes, these chemokines play a key role in the pathogenesis of allergic diseases like atopic dermatitis [15, 16].

TARC/CCL17 is a member of the CC chemokine family and is considered a mediator of the inflammatory responses during the development of inflammatory skin diseases, such as atopic dermatitis [17]. *In vitro* tests using HaCaT cells and human primary keratinocytes and *in vivo* tests using Nc/Nga mice also show that elevated TARC levels when induced by TNF-*α* [18]. MDC/CCL22 is a prototypic chemokine expressed selectively on Th2 cells and intimately involved in Th2-skewed allergic diseases, such as atopic dermatitis [19]. Since MDC is also a member of the Th2-type chemokine family, HaCaT cells express increased MDC level when induced by TNF-*α* and IFN-*γ* [20]. RANTES is a member of a large supergene family of proinflammatory cytokine that plays fundamental role in inflammatory process and expressed in activated T cells, platelets, fibroblasts, airway epithelial cells, or renal epithelial cells [21]. RANTES also acts as a chemotactic signal, attracting monocytes to wound sites [22]. Although RANTES belongs to the Th1-type chemokines, its secretion from HaCaT cells is remarkable when they are stimulated with TNF-*α* and IFN-*γ* [23]. Therefore, the inhibition of TARC, MDC, and RANTES secretion from HaCaT cells is important in relieving the symptoms of allergic diseases.

In this study, we investigated the constituent contents of the reference compounds of Inulae Flos and their inhibitory effects on chemokine production in HaCaT cell treated by extracts prepared with different solvent compositions (70% methanol, 70% ethanol, 100% methanol, 100% ethanol, and water), different solvent fractions (n-hexane, chloroform, ethyl acetate, butanol, and water), and single compounds (chlorogenic acid, caffeic acid, 1,5-dicaffeoylquinic acid, rutin, kaempferol-3-O-glucoside, quercetin, luteolin, and 6-methoxy-luteolin). The 70% methanol extract was deemed to have a better inhibitory effect than extracts produced with other solvents, and this extract was successively partitioned to investigate which fractions contained the most compounds. We found that the ethyl acetate fraction contained all the reference compounds and that the amounts present were higher than in any other fraction, except for caffeic acid, which occurred at higher levels in the water fraction than in any other fraction. 1,5-Dicaffeoylquinic acid occurred at higher levels than the compound in all the fractions. As well as containing constituent compounds than the other extract, the ethyl acetate fraction inhibited the expression of TARC, MDC, and RANTES productions by HaCaT cells better than the other fractions. These results indicated that the ethyl acetate fraction more effectively inhibited the release of chemokines than the other fractions of 70% methanol extract. Therefore, we tentatively inferred that the fraction containing most compounds would maximize the inhibitory effect of Inulae Flos. 

Based on these results, the individual compounds from Inulae Flos were examined to identify which compound inhibited the secretion of chemokines by HaCaT cells. Of the compounds examined, caffeic acid, 1,5-dicaffeoylquinic acid, luteolin, and 6-methoxy-luteolin inhibited the TNF-*α*- and IFN-*γ*-induced expressions of chemokines by HaCaT cells. Out of these compounds, 1,5-dicaffeoylquinic acid which was most abundant both in the 70% methanol extract and in each solvent fraction, especially in ethyl acetate fraction, significantly and dose-dependently inhibited the expression of TARC, MDC, and RANTES in HaCaT cells, whereas the other compounds did not significantly reduce the expression of TARC, MDC, and RANTES in the TI-treated cells. Additionally, luteolin which contained at low level in both 70% methanol extract and ethyl acetate fraction also represented inhibitory effect of TARC, MDC and RANTES productions. 1,5-Dicaffeoylquinic acid is a kind of hydroxycinnamic acid, an ester-formed quinic acid bound by two units of caffeic acid which has anticancer [24] and antioxidant properties [25]. Luteolin is hydroxyflavone-structured compound which blocks mast cell stimulation and T-cell activation in multiple sclerosis [26] and shows diverse biological effect such as antioxidant [27], antitumor [28], and cardio-protective properties [29]. In addition to those biological effects, 1,5-dicaffeoylquinic acid and luteolin can be treated as chemokine-modulator and considered to be closely associated with the inhibition of TNF-*α*- and IFN-*γ*-induced chemokine secretion from HaCaT cells.

## 5. Conclusion

This is the first research to clarify that Inulae Flos has an inhibitory effect on chemokine productions such as TARC, MDC, RANTES in HaCaT cell and its effect could be related with constituent compounds such as 1,5-dicaffeoylquinic acid and luteolin using a method of verification based on successive extracts of the whole herb, fractions of these extracts, and single compounds. We suggest that Inulae Flos containing 1,5-dicaffeoylquinic acid and luteolin can be used as therapeutic agents for allergic disease by inhibiting chemokine production. 

## Figures and Tables

**Table 1:** Linear regression, correlation coefficients (*r* ^2^), LOD, and LOQ for the reference compounds (*n* = 3).

Compound	Regression equation	Correlation coefficient (*r* ^2^)	Linear range (*μ*g/mL)	LOD (*μ*g/mL)	LOQ (*μ*g/mL)
Chlorogenic acid	*y* = 32900*x* – 11317	0.9997	1.56–50	0.03	0.09
Caffeic acid	*y* = 52440*x* – 4993.7	0.9999	0.63–20	0.02	0.06
1,5-Dicaffeoylquinic acid	*y* = 27029*x* – 115613	0.9996	12.50–200	0.13	0.43
Rutin	*y* = 18447*x* – 3067.7	0.9999	1.56–50	0.05	0.16
Kaempferol-3-O-glucoside	*y* = 21596*x* – 1615.6	0.9998	0.31–5	0.06	0.20
Quercetin	*y* = 28313*x* – 10729	0.9995	0.63–20	0.05	0.15
Luteolin	*y* = 39978*x* – 13399	0.9998	0.78–25	0.03	0.10
6-Methoxy-luteolin	*y* = 27712*x* – 10638	0.9996	0.78–25	0.04	0.13

LOD: limit of detection; LOQ: limit of quantification; *y*: peak area (mAU); *x*: concentration of compound (*μ*g/mL).

**Table 2:** Intraday and interday precision of the reference compounds.

Compound	Spiked concentration (*μ*g/mL)	Intraday (*n* = 3)		Interday (*n* = 3)	
		Detected concentration (*μ*g/mL)	RSD (%)	Detected concentration (*μ*g/mL)	RSD (%)
	3.5	3.42	0.35	3.41	0.18
Chlorogenic acid	7	7.30	0.42	7.37	0.53
	10	9.82	0.23	9.77	0.29

	1.5	1.44	0.45	1.45	0.67
Caffeic acid	3	3.04	0.70	3.05	0.72
	4.5	4.49	0.31	4.49	0.34

	35	34.87	3.24	35.55	0.56
1,5-Dicaffeoylquinic acid	70	70.91	0.65	71.05	0.31
	100	99.41	0.72	99.08	0.14

	5	5.15	1.03	5.18	1.48
Rutin	10	10.28	1.03	10.24	1.23
	15	14.77	0.36	14.78	0.40

	0.4	0.38	1.25	0.38	1.28
Kaempferol-3-O-glucoside	0.8	0.79	2.11	0.79	2.60
	1	1.02	1.42	1.00	2.02

	2	2.09	2.11	2.09	2.15
Quercetin	4	3.91	1.00	3.90	1.47
	6	6.03	0.25	6.03	0.43

	1.3	1.29	2.92	1.29	2.42
Luteolin	2.5	2.47	1.41	2.42	1.62
	3.5	3.53	0.37	3.56	1.09

	1.5	1.47	1.51	1.45	0.92
6-Methoxy-luteolin	3	2.88	1.40	2.89	0.16
	4	4.10	0.85	4.11	0.04

RSD: relative standard deviation (%) = (standard deviation/mean) × 100.

**Table 3:** Recovery of the reference compounds (*n* = 3).

Compound	Initial concentration (*μ*g/mL)	Spiked concentration (*μ*g/mL)	Detected concentration (*μ*g/mL)	Recovery (%)	RSD (%)
		3.5	3.65	104.23	0.71
Chlorogenic acid	9.75	7	7.78	111.13	0.35
		10	10.36	103.65	1.77

		1.5	1.56	103.68	2.90
Caffeic acid	3.74	3	3.28	109.29	1.41
		4.5	4.85	107.71	1.50

		35	34.95	99.85	2.36
1,5-Dicaffeoylquinic acid	78.08	70	71.16	101.66	1.19
		100	98.19	98.19	0.44

		5	5.28	105.64	0.89
Rutin	10.77	10	10.49	104.92	1.82
		15	14.73	98.23	0.86

		0.4	0.40	99.60	1.66
Kaempferol-3-O-glucoside	0.86	0.8	0.83	103.36	1.75
		1	1.06	105.86	2.05

		2	2.05	102.51	2.47
Quercetin	4.10	4	3.86	96.52	0.87
		6	5.90	98.27	1.08

		1.3	1.33	102.32	1.56
Luteolin	1.94	2.5	2.49	99.67	1.49
		3.5	3.56	101.74	1.03

		1.5	1.41	93.88	1.20
6-Methoxy-luteolin	2.86	3	2.79	93.09	0.43
		4	4.00	99.95	2.55

**Table 4:** Average contents of the reference compounds in extracts produced with different solvent compositions (*n* = 3).

Compound	Average content in each different solvent composition (mg/g)^a^				
	70% methanol	70% ethanol	Methanol	Ethanol	Water
Chlorogenic acid	7.27 ± 0.18	5.89 ± 0.19	3.57 ± 0.09	1.49 ± 0.00	0.34 ± 0.02
Caffeic acid	2.95 ± 0.01	2.47 ± 0.01	3.00 ± 0.01	1.40 ± 0.02	20.51 ± 1.89
1,5-Dicaffeoylquinic acid	59.69 ± 1.41	49.18 ± 1.53	33.23 ± 0.84	19.23 ± 0.31	20.86 ± 1.19
Rutin	6.34 ± 0.56	5.03 ± 1.06	7.44 ± 0.12	5.56 ± 0.21	0.43 ± 0.03
Kaempferol-3-O-glucoside	0.63 ± 0.02	0.34 ± 0.02	0.81 ± 0.01	0.65 ± 0.04	0.27 ± 0.01
Quercetin	2.97 ± 0.13	4.09 ± 0.09	5.96 ± 0.12	8.26 ± 0.24	ND
Luteolin	2.13 ± 0.04	1.96 ± 0.03	3.25 ± 0.03	4.35 ± 0.04	0.33 ± 0.02
6-Methoxy-luteolin	3.01 ± 0.14	2.68 ± 0.10	4.47 ± 0.05	5.77 ± 0.17	0.42 ± 0.00

ND: not detected.

^a^Average content represented as mean ± SD.

**Table 5:** Average contents of the reference compounds in the solvent fractions of the 70% MeOH extract (*n* = 3).

Compound	Average content in each solvent fraction (mg/g)^a^				
	n-Hexane	Chloroform	Ethyl acetate	Butanol	Water
Chlorogenic acid	ND	ND	2.47 ± 0.04	16.77 ± 0.21	2.05 ± 0.06
Caffeic acid	ND	0.12 ± 0.00	14.06 ± 0.10	1.34 ± 0.02	0.22 ± 0.00
1,5-Dicaffeoylquinic acid	ND	4.57 ± 0.02	248.79 ± 2.62	41.09 ± 0.39	4.43 ± 0.01
Rutin	0.28 ± 0.01	0.32 ± 0.03	56.00 ± 0.02	5.73 ± 0.14	ND
Kaempferol-3-O-glucoside	ND	ND	3.67 ± 0.22	0.20 ± 0.03	ND
Quercetin	ND	ND	23.02 ± 0.24	0.94 ± 0.02	ND
Luteolin	ND	ND	12.88 ± 0.22	ND	ND
6-Methoxy-luteolin	ND	1.33 ± 0.04	15.39 ± 0.16	ND	ND

ND: not detected.

^a^Average content represented as mean ± SD.
